# Management of saltwater intrusion using 3D numerical modelling: a first for Pacific Island country of Vanuatu

**DOI:** 10.1007/s10661-023-12245-y

**Published:** 2024-01-09

**Authors:** Ashneel Sharan, Bithin Datta, Alvin Lal, Krishna K. Kotra

**Affiliations:** 1 Discipline of Civil Engineering, College of Science & Engineering, James Cook University, Townsville, QLD 4811 Australia; 2 Global Centre for Environmental Remediation, College of Engineering, Science and Environment, University of Newcastle, Callaghan, NSW 2308 Australia; 3 crcCARE, The University of Newcastle, Callaghan, NSW 2308 Australia; 4 School of Agriculture, Geography, Environment, Oceans & Natural Sciences, The University of the South Pacific, Emalus Campus, Port Vila, Vanuatu

**Keywords:** Groundwater modelling, Saltwater intrusion, SEAWAT, Coastal aquifer, Hydraulic barriers

## Abstract

Small island countries like Vanuatu are facing the brunt of climate change, sea level rise (SLR), tropical cyclones, and limited or declining access to freshwater. The Tagabe coastal aquifer in Port Vila (the capital of Vanuatu) shows the presence of salinity, indicating saltwater intrusion (SWI). This study aims to develop and evaluate effective SWI management strategies for Tagabe coastal aquifer. To manage SWI, the numerical simulation model for the study area was developed using the SEAWAT code. The flow model was developed using MODFLOW and the transport model was developed using MT3DMS. Whereby SEAWAT solved flow and transport equations simultaneously. The model was calibrated, and different scenarios were evaluated for the management of SWI. The SLR was also considered in the model simulations. The results indicated that increased population, pumping rates, and SLR affect the SWI rates. To manage the SWI, we introduced hydraulic barriers like barrier wells and injection wells which effectively managed SWI in Tagabe coastal aquifer. The results from this study are significantly important whereby, the water managers, site owners, and governing bodies can use the management strategies presented in this study to create policies and regulations for managing SWI rates in Port Vila. Additionally, the water industry, private businesses, and investors who wish to extract groundwater from the Tagabe can use this study as a reference for daily or yearly freshwater production rates without the risk of SWI.

## Introduction

**Fig. 1:**
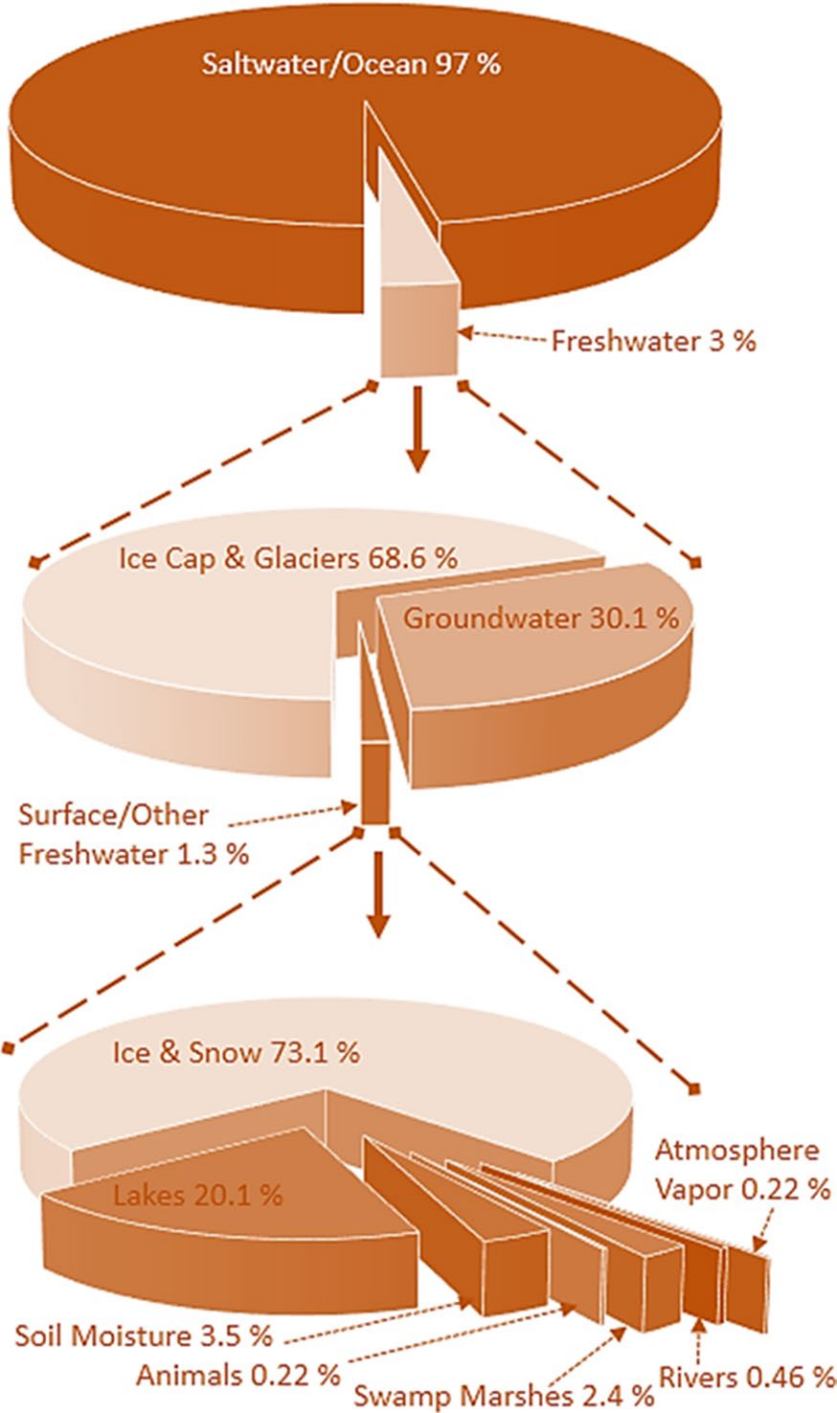
Earth’s water resources. Source (Shiklomanov, 1993)

Water resources are fundamental for the sustainability and survival of all living beings on Earth and beyond. This precious commodity is declining; only 3% of all water is freshwater, and out of this 3%, 31.4% is usable, whereas 68.6% are ice caps and glaciers. Moreover, groundwater reserves are approximately 30.1%, whereas surface water sources are 1.3% (Shiklomanov, 1993). The percentage of water resources remaining on our planet is shown in Fig. 1. From the graph, it can be shown that the majority of the freshwater available is groundwater. However, these groundwater resources are also constantly threatened by natural and anthropogenic means (Khatri & Tyagi, 2015; Li et al., 2021; Sharan et al., 2023a, b; Vengadesan & Lakshmanan, 2019).

Groundwater is the primary water source for the Pacific Island countries (PICs) (Sharan et al., 2021). Almost all PICs use groundwater for their daily needs, which puts groundwater under threat. The major environmental threat of coastal aquifers in PICs is saltwater intrusion (SWI) (Kotra et al., 2022). SWI occurs when the seawater moves towards the landward side, changing the freshwater-saltwater pressure gradients. The SWI is an environmental threat in PICs and all around the globe in coastal regions (ACASA, 2011). The management of SWI is crucial, especially in small island countries which are predominantly surrounded by ocean. The SWI management in PICs is minimal due to the unavailability of expertise, proper groundwater data management, and unavailability of specialised monitoring devices (Sharan et al., 2021). Very few PICs thoroughly monitor and collect accurate groundwater data, for instance, Kiribati (1.84° N, 157.38° W) (Lal & Datta, 2019c). The coastal aquifers in Kiribati have been extensively studied with some numerical and AI-based modelling techniques that provide optimal SWI management strategies (e.g. Lal & Datta, 2019a, b). Other limited studies conducted in PICs include Nauru (0.52° S, 166.93° E) (Alberti et al., 2017) and Tonga (21.18° S, 175.19° W) (Sharan et al., 2023a, b; White et al., 2011).

The use of numerical-based modelling techniques is complex and computationally expensive. However, they are mainly the first source of SWI management tools in groundwater. Later comes the AI-based modelling techniques, utilising the results from numerical models. Moreover, without the input–output patterns from a numerical model, one cannot develop accurate AI-based models. Therefore, numerical models are crucial for developing curative solutions to reduce SWI (Hamidi et al., 2021). Numerical modelling has been extensively applied to manage SWI around the globe (e.g. Andersen et al., 1988; Bobba, 2002; Dhar & Datta, 2009; Giambastiani et al., 2007; Lee et al., 2023; Stein et al., 2023). All these studies used numerical modelling techniques to provide optimal SWI management strategies for coastal aquifers. Numerical models are mainly developed using the finite element method (FEM) or finite difference method (FDM). However, the models developed using the latter methods use grids which makes it easier for optimum SWI management scenario testing (Sharan et al., 2023a, b; Werner et al., 2013). Different codes can be used in FDM for modelling groundwater flow and transport, for example, MODFLOW, MT3DMS, PHT3D, and SEAWAT.

SEAWAT numerical codes have been relatively applied in managing the SWI in coastal aquifers around the globe. Lin et al. (2009) used the SEAWAT code to develop a numerical simulation model to investigate the amount of SWI in the Gulf Coast aquifers of Alabama (32.31° N, 86.90° W), USA. The authors calibrated and validated their model using the hydraulic head values. The calibrated model was used to predict the future SWI in Alabama. The authors reported that the extent of SWI has increased during a 40-year simulation period. The authors also stated that due to population growth and more water demands, the SWI would be more severe in the future. Romanazzi et al. (2015) developed a numerical simulation model for managing a Mediterranean karstic coastal aquifer affected by SWI and climate change. The authors used the SEAWAT code to develop their model and used the hydraulic head and the salinity data to calibrate and validate the model. The calibrated model was simulated to predict three future scenarios: climate change, different types of discharge, and SLR. The authors reported that all the scenarios tested showed non-negligible effects on coastal groundwater. The authors also suggested that to manage SWI, the current water technologies must be modified, water to be recycled, and aquifers must be artificially recharged.

Casillas-Trasvina et al. (2019) applied the SEAWAT code to develop a numerical simulation model to assess multi-source SWI under natural and pumping conditions in the Great Maputo aquifer (25. 97° S, 32.57° E), Mozambique. The authors used hydraulic heads, discharges, and salt concentrations to calibrate and validate the model. The calibrated model was used to test different scenarios, including the impacts of SLR and projected abstraction rates. The results indicated that SWI is higher for the scenarios tested. The authors also reported that management strategies must be drawn, and using a more significant number of scattered production wells that can be operated with lower pumping rates will prevent the saltwater upconing. Abd-Elhamid et al. (2020) provided the SWI management strategies for Biscayne aquifer in Florida, USA, using numerical modelling. The authors suggested that extending the coastline towards the sea using coastal earth-fill would increase the land surface area and reduce SWI rates. However, this technique of managing SWI could damage the existing coastal ecosystems and marine organisms. Also, this land reclamation technique could be very costly and could have low hydraulic conductivity for reclaimed soil.

Dibaj et al. (2020) developed a numerical model using SEAWAT to simulate the SWI in the Pingtung coastal aquifer (22.70° N, 120.44° E) in Taiwan under the influence of SLR and changing abstraction regimes. The model was calibrated and validated using the observed head values. The authors used the calibrated model to predict the future SWI into the aquifer. Three management scenarios were examined, including the SLR, pumping rates, and relocation of production wells. The authors reported that all three scenarios affect the extent of SWI in their study area. Moreover, all the layers of the aquifer were affected by SWI. However, the middle layer was least affected by SWI when compared with the top and bottom layers.

Said and Yurtal (2022) used the SEAWAT code to develop a numerical simulation model to predict the SWI in the coastal aquifer of Bosaso Plain (11. 28° N, 49.19° E), Somalia. The authors calibrated and validated the model using the hydraulic head and total dissolved solid concentrations. The calibrated model was used to predict SWI by changing pumping patterns. The authors reported that SWI mainly affects the coastline, and increasing the pumping will cause SWIs to reach 6.2 km inland by 2040. Moreover, the authors also reported that reducing the pumping rates by 50% will reduce SWI significantly. Abd-Elaty & Polemio (2023) used the SEAWAT numerical code to provide SWI management strategies for the Gaza Strip (32.35° N, 34.31° E) aquifer in Palestine. The authors used the SLR and the reduction in fresh groundwater storage to show SWI impacts. The authors found that SWI was more severe if the SLRs and pumping increased. They suggested that using cutoff wells and check dams effectively managed SWI.

The majority of the studies mentioned earlier have used different scenarios in testing and mitigating the SWI. However, we will be using a different approach. The use of a numerical simulation model to manage SWI in PICs is minimal, which forms the central focus area of this research. The present study tends to develop first-ever SWI management techniques using 3D numerical simulation models for Vanuatu (15.38° S, 166.96° E) in the South Pacific region. To the best of our knowledge, no study has been conducted in Vanuatu that safeguards groundwater or provides SWI management strategies. In this study, the increase in population and pumping rates will be raised in transient mode gradually. The standing water levels or the hydraulic head values are also raised gradually to factor in the SLR with time. The combination of barrier well and injection well will also be modelled in this study.

The knowledge gained from this study is novel and of foremost importance for Vanuatu and all other surrounding island nations in the Pacific region, as it will provide optimal SWI management strategies by considering uncertainties, including climate change. Moreover, as mentioned above, the scenarios evaluated are the first for an island nation in the Pacific region, making this study necessary for the smaller island nations. The results are beneficial for drawing up robust SWI management strategies in Vanuatu. These strategies will help protect groundwater resources for future use and reduce saline water contaminations. In addition, this study also validates strategies that could be used to increase the abstraction rates and simultaneously restrict SWI into the coastal aquifers of Vanuatu. Finally, this study provides new knowledge to stakeholders and/or bore drilling companies who want to harvest groundwater in Vanuatu, as it gives them the benchmark of fresh groundwater’s daily or yearly production rates.

## Materials and methods

### Study area

#### ***Country profileand geology***

Vanuatu is located on the eastern side of Australia and comprises eighty-three islands, some volcanic and mountainous, while others are coral atolls. The country has little over 300,000 people, and its capital is Port Vila. Vanuatu is known for its stunning natural beauty, with lush rainforests, pristine beaches, and clear waters. The country is a popular destination for tourists who enjoy snorkelling, scuba diving, and hiking in the mountains. According to a report by the World Travel and Tourism Council (WTTC), tourism significantly contributes to Vanuatu’s economy, amounting to 35.5% of its GDP in 2019 (WTTC, 2021). In addition to tourism, Vanuatu’s economy is also supported by agriculture, fishing, and forestry. The country is known for producing tropical fruits, such as coconuts, bananas, and pineapples, as well as coffee and cocoa beans.

Vanuatu is a culturally diverse nation, with over one hundred indigenous languages spoken across its islands. The country has a rich cultural heritage, with traditional practices such as custom dancing and sand drawing still practised today. Vanuatu’s culture and traditions have been recognised by UNESCO, which has designated several sites in the country as World Heritage Sites, including the Chief Roi Mata’s Domain and the Cultural Centre in Port Vila (UNESCO, 2021). However, like many small island nations, Vanuatu is vulnerable to natural disasters and the impacts of climate change. The country has experienced severe tropical cyclones, volcanic eruptions, and earthquakes in recent years, which have caused considerable damage to infrastructure and livelihoods (Sharan, 2022, 2023; VMGD, 2023). The government of Vanuatu has recognised the importance of building resilience to these threats and has taken steps to incorporate climate change adaptation and disaster risk reduction measures into its development planning (CRCP, 2021; SPC, 2015).

The country is divided into six provinces: Malampa, Penama, Sanma, Shefa, Tafea, and Torba. The province Shefa is in the country’s centre, consisting of two major islands, Epi and Efate. Port Vila is the capital of Vanuatu, located on Efate Island, with an area of approximately 980 km^2^. The primary focus of this research is based on Efate Island. Efate has diverse geology, including volcanic rocks, limestones, and alluvial deposits, which several geological processes have shaped. In addition to volcanic rocks, the island has several limestone and alluvial deposits. The limestone deposits are in the central and eastern parts of the island and were formed from the accumulation of marine organisms such as corals and shells.

Furthermore, the alluvial deposits are in the coastal plains and were formed by the erosion and deposition of sediment from nearby rivers and streams (Bath et al., 1986). Several geological processes, including volcanic activity, tectonic movements, and erosion, have also shaped the island. The volcanic activity on the island has created several volcanic features, including the Yasur volcano, one of the most active and accessible volcanoes in the world (Pfeiffer, 2023). Tectonic movements have also shaped the island’s topography, with several faults and folds. Erosion has also played a significant role in shaping the island’s landscape, with the coastal areas being highly susceptible to erosion due to their low elevation and proximity to the sea (Stewart et al., 2010).

#### Groundwater site hydrogeology

**Fig. 2:**
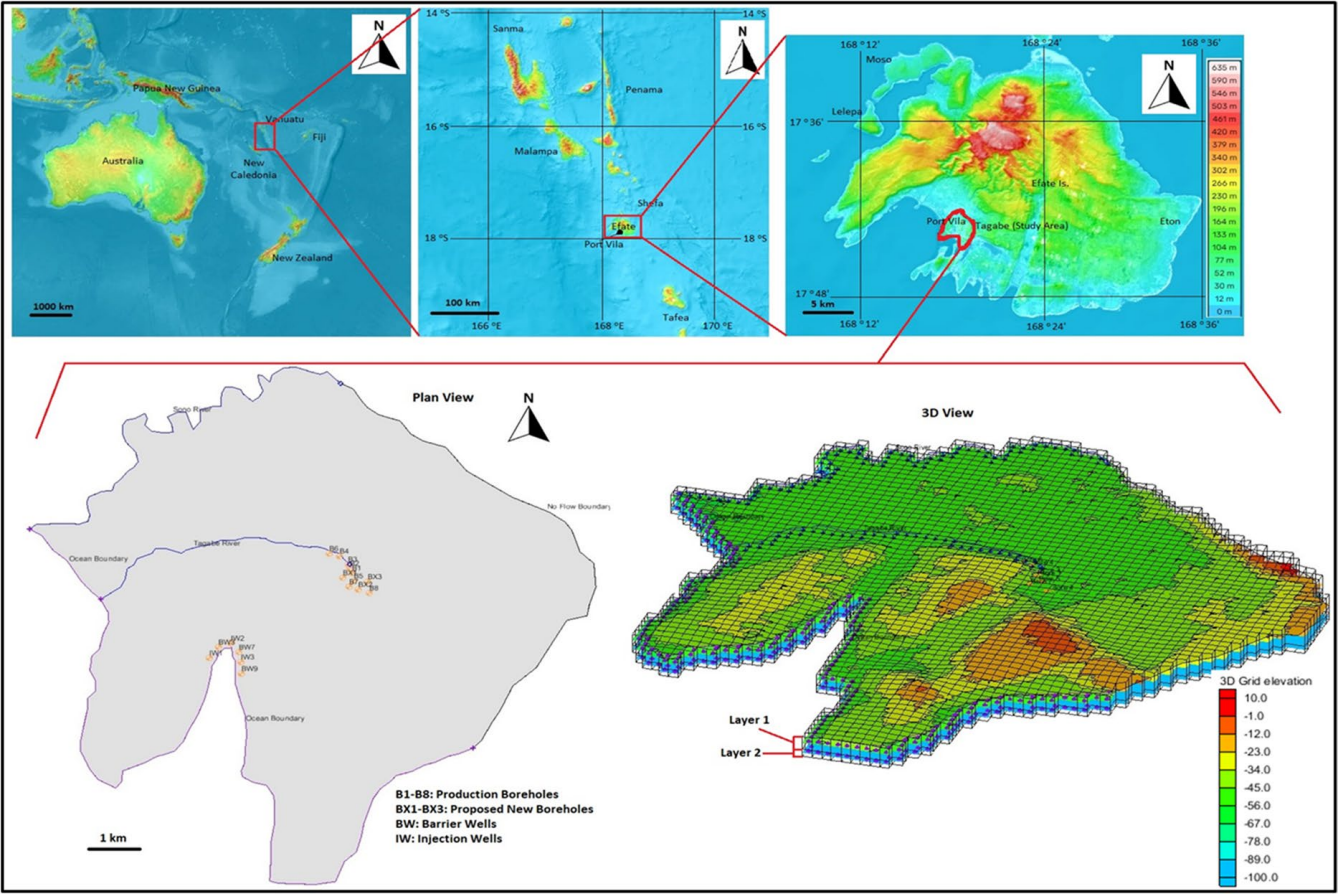
Study area inclusive of the plan and 3D views

The water supply in Port Vila comes from the Tagabe catchment (17.70° S, 168.32° E). The study area for this research is Tagabe coastal aquifer, shown in Fig. 2. The hydrogeology of this area is complex and influenced by the island’s geology, rainfall patterns, and land use. According to a report by the Vanuatu Geohazards Observatory (VGO), the Tagabe catchment area has a fractured volcanic rock aquifer system composed of basalts and andesites (VGO, 2015). A study by the United Nations Development Programme (UNDP) identified several factors affecting groundwater recharge in the Tagabe Catchment area, including rainfall patterns, land use, and soil characteristics (UNDP, 2016). Tagabe catchment supplies water to the residents, businesses, agriculture, and manufacturing industries in Port Vila. However, with the rapid urbanisation and population growth of Port Vila and the push to increase tourism and infrastructure, there will be a higher demand for freshwater per person, putting more pressure on the Tagabe freshwater aquifer. Climate change will also make Port Vila and Vanuatu more vulnerable, further jeopardising the ability to support current and future users of the Tagabe water (TRCMP, 2018).

Tagabe catchment has eight active production boreholes which supply water in Port Vila. The log of borehole EF378 data from 1994 suggested that the Tagabe aquifer can be considered two layers. The first layer has sand and gravel, whereas the second is mainly limestone. The depth of layer one goes to 80 m, and then limestone is below that. Therefore, to cater for any uncertainties, we just considered 20 m of limestone as layer 2. Moreover, the Tagabe coastal aquifer is heterogeneous, shallow, and unconfined. Tagabe coastal aquifer has two active rivers, Sono and Tagabe river, which also acts as a source of the Tagabe aquifer. The area we have modelled is approximately 57 km^2^, with eight boreholes operating in close proximity.

The Department of Water Resources, Government of Vanuatu, supplied the groundwater data. However, the data was limited. The dataset included bore pumping data from 2017 to 2022 and the electrical conductivity of some bores from 2019–2021. The hydraulic head values were not available, nor were the initial concentrations. We have utilised the limited data to develop our numerical model, calibrate and validate, and test different scenarios that can manage SWI in Tagabe coastal aquifer. The hydrogeological parameters and other boundary conditions were taken from the bore log data and the report by Depledge (1994). The report can be shared with the mutual consent from the Government of Vanuatu. The report mentioned that hydraulic conductivity was between 20 m/d and 45 m/d based on the soil types. Moreover, the specific storage value of approximately 0.000035 1/m was assumed based on the limited data from the report. Vanuatu receives a lot of rain and 90% can be runoff. Therefore, we have used 7.5% of the rainfall as recharge and 3% as evapotranspiration. Other hydrogeological parameters and boundary conditions used from the field are given in Table 1.

**Table 1:**
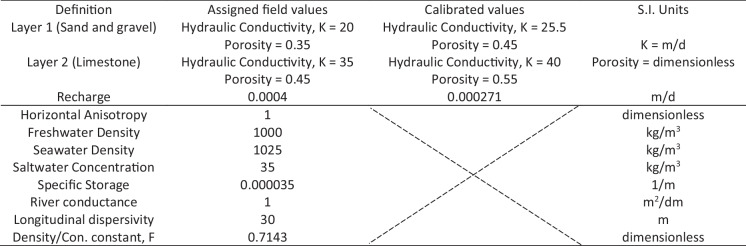
Model parameters and the values used for the model design

### Flow and solute transport model

Developing management strategies for SWI is a complicated and nonlinear process that can be addressed using mathematical models such as numerical simulation models. In order to simulate and manage SWI, a numerical simulation model was developed using SEAWAT code within the Groundwater Modeling System (GMS). SEAWAT uses a numerical code to replicate 3D, variable density, and transient groundwater flow in porous mediums by combining the MODFLOW and MT3DMS programs. MODFLOW solves the nonlinear variable density flow equations, while MT3DMS addresses the nonlinear temporal and spatial salt or solute transport equations, as described by Guo and Langevin (2002).

#### Governing equations

The variable-density flow process solves the following form of the variable-density groundwater flow equation (tensors and vectors are shone in bold) (Langevin et al., 2008).

1 $$\nabla \bullet \left[\rho \frac{{\mu }_{0}}{\mu }{\mathbf{K}}_{0}\left( \nabla {h}_{0}+ \frac{\rho - {\rho }_{0}}{{\rho }_{0}} \nabla z\right)\right]= \rho {S}_{s,0} \frac{\partial {h}_{0}}{\partial t}+ \theta \frac{\partial \rho }{\partial C} \frac{\partial C}{\partial t}- {\rho }_{s}{q}_{s}{\prime} ,$$

where ${\rho }_{0}$ is the fluid density [kg/m^3^] at the reference concentration and reference temperature, *μ* is the dynamic viscosity [kg/md], **K**_**0**_ is the hydraulic conductivity tensor of material saturated with the reference fluid [m/d], *h*_*0*_ is the hydraulic head [m] measured in terms of the reference fluid of a specified concentration and temperature (as the reference fluid is commonly freshwater), *S*_*s,0*_ is the specific storage [1/m], defined as the volume of water released from storage per unit volume per unit decline of *h*_*0*_, *t* is time [d], $\theta$ is porosity [dimensionless], *C* is salt concentration [kg/m^3^], and *q'*_*s*_ is a source or sink [1/d] of fluid with density ${\rho }_{s}$.

The integrated MT3DMS Transport process solves the following form of the solute transport equation.

2 $$\left(1+ \frac{{\rho }_{b}{K}_{d}^{k}}{\theta } \right)\frac{\partial ( \theta {C}^{k} )}{\partial t}=\nabla \bullet \left(\theta \mathbf{D}\bullet \nabla {C}^{k} \right)- \nabla \bullet \left(\mathbf{q}{C}^{k}\right)- {q}_{s}{\prime} {C}_{s}^{k}+{\sum }_{k=1}^{N}{R}_{k}$$

where ${\rho }_{b}$ is the bulk density (mass of the solids divided by the total volume) [mg/m^3^], ${K}_{d}^{k}$ is the distribution coefficient of species *k* [m^3^/kg], ${C}^{k}$ is the concentration of species *k* [kg/m], **D** is the hydrodynamic dispersion coefficient tensor [m^2^/d], **q** is specific discharge [m/d], and ${C}_{s}^{k}$ is the source or sink concentration [kg/m^3^] of species *k*. ${R}_{k}$ (*k* = *1, …N*) is the chemical reaction term (kg/m^3^d) of species *k.*

SEAWAT’s earlier versions assumed that $\frac{{\mu }_{0}}{\mu }$ was equal to one, resulting in viscosity effects being ignored and fluid density being considered as a simple linear function of only one solute species. In SEAWAT version 4, fluid density and viscosity can be computed using concentrations from one or more solute species. These equations of state link the density and viscosity terms in Eq. 1 to one or more of the MT3DMS species concentrations (*C *^*k*^) in Eq. 2. By permitting one or more MT3DMS species to affect fluid density and viscosity, variable-density groundwater flow can be paired with simultaneous solute and heat transport (Langevin et al., 2008). The previous edition of SEAWAT and the variable density groundwater flow and transport equations can be found in the studies by Guo and Langevin (2002) and Zheng and Wang (1999).

#### Conceptual and flow model design

The MODFLOW-2000 code was used to develop the conceptual model of the Tagabe coastal aquifer. The length in the model domain’s X, Y, and Z directions were 7.50 km, 7.61 km, and 0.1 km, respectively. A 100 × 100 m cell size was used to create 3D finite-difference grids for the entire model domain. Two layers were created with different values of hydraulic conductivities in both layers. The uniform hydraulic conductivities of layers 1 and 2 were assigned as 20 m/d and 35 m/d, respectively. The porosities of layer 1 and layer 2 were also assigned as 0.35 and 0.45, respectively. Layer 1 was from 0 to − 80 m, while layer 2 was from − 80 to − 100 m. The southern part of the model domain was the specified head boundary (CHB), while the eastern side was no flow boundary. The northwestern side was the Sono River, connected to the ocean boundary and no flow boundary. We have assigned no flow boundary merely because the groundwater flows parallel to the no-flow boundary. Tagabe River is connected to the ocean boundary from the southwestern side and goes up to the middle of the model domain. Tagabe River is near the production bore and the Vanuatu International Airport.

The observed hydraulic head values from the boreholes in Tagabe were not available. However, only one value of each borehole was provided, which was recorded in 2019. Approximate head values were assigned to the boundaries using the given head values. The CHB values for the western and southeastern nodes were assigned as 9 m and 8.8 m, respectively. The head values were assigned in such a way that the hydraulic gradient was slightly towards the landward side. This was done because the initial conditions and head values were unknown, but the salinity was present in the Tagabe coastal aquifer, indicating a landward-side hydraulic gradient.

Moreover, the head values of the Sono River for the western and northern nodes were assigned as 9.2 m and 9 m, respectively. At the same time, the head values for Tagabe River at the western and in the centre of the model domain nodes were assigned as 9.2 m and 7 m, respectively. All eight boreholes (B1–B8) were added to the model domain using their specific geographic locations from Table 2. The actual transient pumping values from 2017 to 2021 were also assigned to each bore. Furthermore, all the hydrogeological parameters and boundary conditions, wells, pumping values, and head values were mapped to MODFLOW, and the steady-state simulation was carried out for 20 years to establish the initial conditions.

**Table 2:** Location of production boreholes in Tagabe, Port Vila

Borehole number	Location	
	Latitude (S)	Longitude (E)
B1	17.710828	168.323633
B2	17.710253	168.323403
B3	17.709753	168.323186
B4	17.708767	168.322242
B5	17.711758	168.323814
B6	17.708433	168.321072
B7	17.712467	168.323269
B8	17.713261	168.325547

#### Solute transport model design

Upon successful convergence of the flow model, the solute transport solver, MT3DMS, was introduced, and the simulation was changed from a steady state to a transient state by adding the 5-year simulation periods. The head values from the steady-state simulations were used as initial heads in the transient flow simulations. The solute salt was added to MT3DMS, and a uniform initial salt concentration of 0.1 kg/m^3^ was assigned in the model domain. The advection, dispersion, source, and sink mixing packages were used for solute transport in MT3DMS. SEAWAT-version 4 code was then introduced to solve the solute transport equations with the help of MT3DMS. A variable-density flow package was utilised to solve solute transport for freshwater and saltwater fluids.

Moreover, in the SEAWAT module, freshwater density was selected as the standard fluid density. A constant factor, F, was also applied to the density and concentration slope to prevent the model from overestimating the freshwater lens. If F were set to zero, then the density would not change with a change in concentration, providing similar results to those obtained from MT3DMS simulations. However, SEAWAT was preferred for generating results as it incorporates a variable-density flow (VDF) that restricts the free movement of freshwater and seawater, forming a transitional zone for the freshwater lens.

### Model calibration and validation

SEAWAT was initiated for the 5-year period considering the pumping data from 2017 to 2021. However, we only had the electrical conductivity (EC) data of boreholes B1, B2, B3, B7, and B8 to calibrate the model. The EC data was only available from 2019 to 2021. However, some data was missing from the year 2021. Hence, we only used 2019 and 2020 EC data to calibrate the model. EC was converted to parts per million (ppm) or milligrams/litre (mg/L) by multiplying the EC, measured in microseimens/centimetre (μS/cm), values by 0.55 (Landscape-SA., 2015; Rusydi, 2018). Then, we divided mg/L by 1000 to convert the salt concentration to kg/m^3^. The limitation of this calibration process was that the flow model was not calibrated because we did not have observed head values. Hence, we only used secondary data in the calibration process. Calibrating the model with secondary data was difficult and time-consuming, especially when the initial concentrations were unknown. However, using the trial-and-error method and multiple simulations, we adjusted the initial salt concentrations, recharge value, hydraulic conductivities, and porosities to get the desired results.

After calibrating the model, we simulated the model for the next 20 years, from 2022 to 2043. The results from the calibrated model became the base case for this study. Using the same calibrated model and stress period, we tested and simulated various scenarios that can manage the SWI for Tagabe coastal aquifer.

### Simulated scenarios

Once the numerical simulation model was developed, calibrated, and validated, the base case simulation was run for 20 years to see the extent of SWI into the Tagabe coastal aquifer. The simulated results indicated extensive SWI. Therefore, we evaluated scenarios that can manage SWI considering uncertainties like climate change. The scenarios included (1) an increase in population and pumping rates, (2) the use of barrier wells, (3) the use of barrier wells plus injection wells, and (4) a sea level rise. These scenarios were compared with the base case, and the optimal SWI management strategies were suggested for Tagabe coastal aquifer.

## Results

### Calibration and validation

**Fig. 3:**
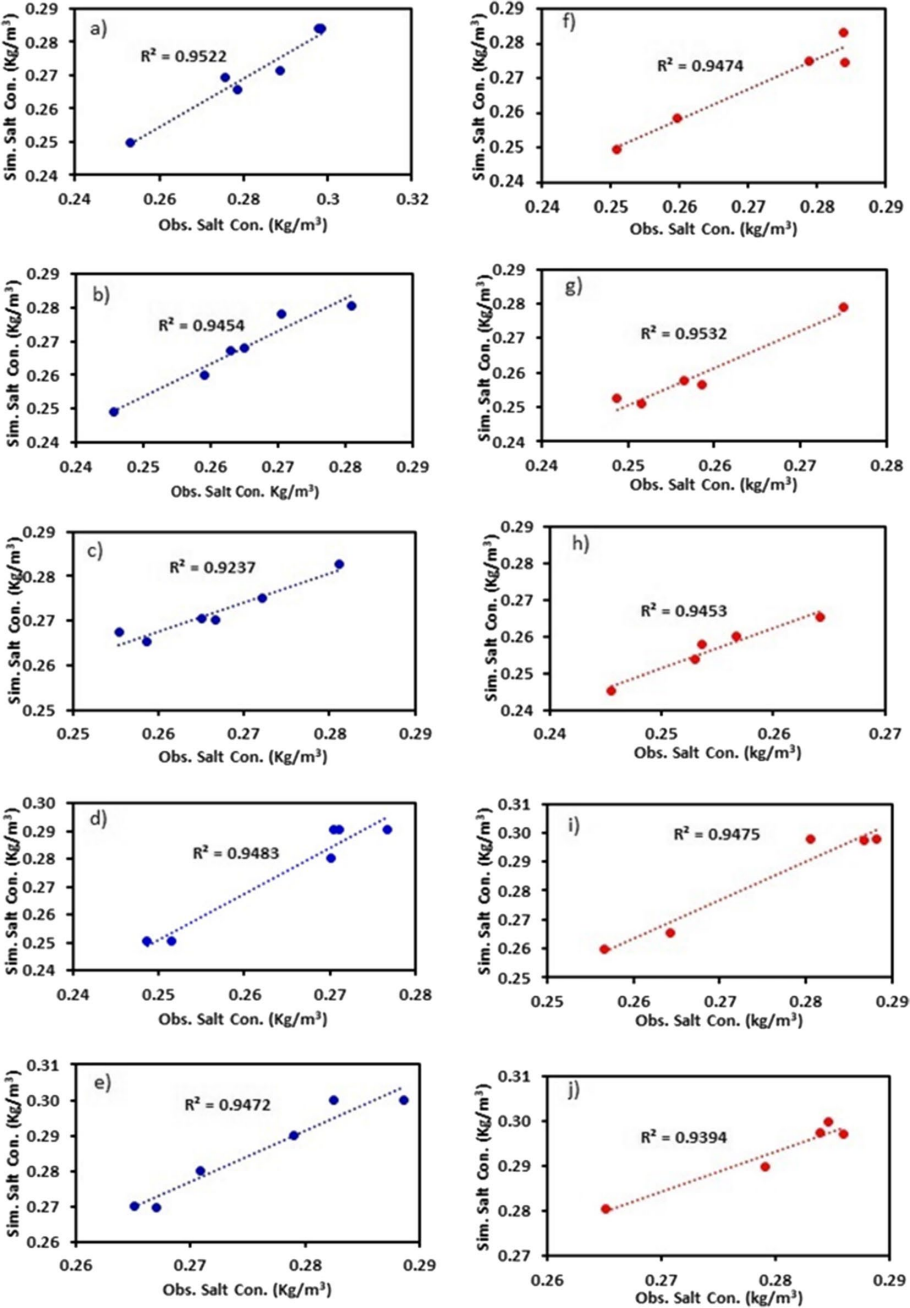
Observed versus simulated salt concentrations obtained during calibration (**a–e**) and validation stage (**f–j**). **a–e** represent the data from bores 1, 2, 3, 7, and 8, respectively. Similarly, **f–j** represent the data from bores 1, 2, 3, 7, and 8, respectively

The model was calibrated using the techniques mentioned in “Scenario 1: increased population and pumping rates.” The observed salt concentrations of March, May, July, September, November 2019, and February 2020 were used in the calibration process. The months of April, June, August, October, and December 2020 were used to validate the model. These months were chosen based on the quality and availability of the data points. The calibration and validation results of different boreholes are shown in Fig. 3. The initial salt concentration that gave the desired results was 0.3 kg/m^3^. The model was simulated for 5 years (2017–2021). However, the calibration started from 3rd year (2019), which gave us a good correlation (*R*^*2*^ > 0.9) between observed and simulated values because the initial concentration was automatically readjusted based on transient pumping rates. If we had complete data, calibration would have started using the data from 2017. Therefore, the initial concentrations had to be assigned as < 0.3 kg/m^3^. The calibrated model was then used to test different scenarios.

### Base case

**Fig. 4:**
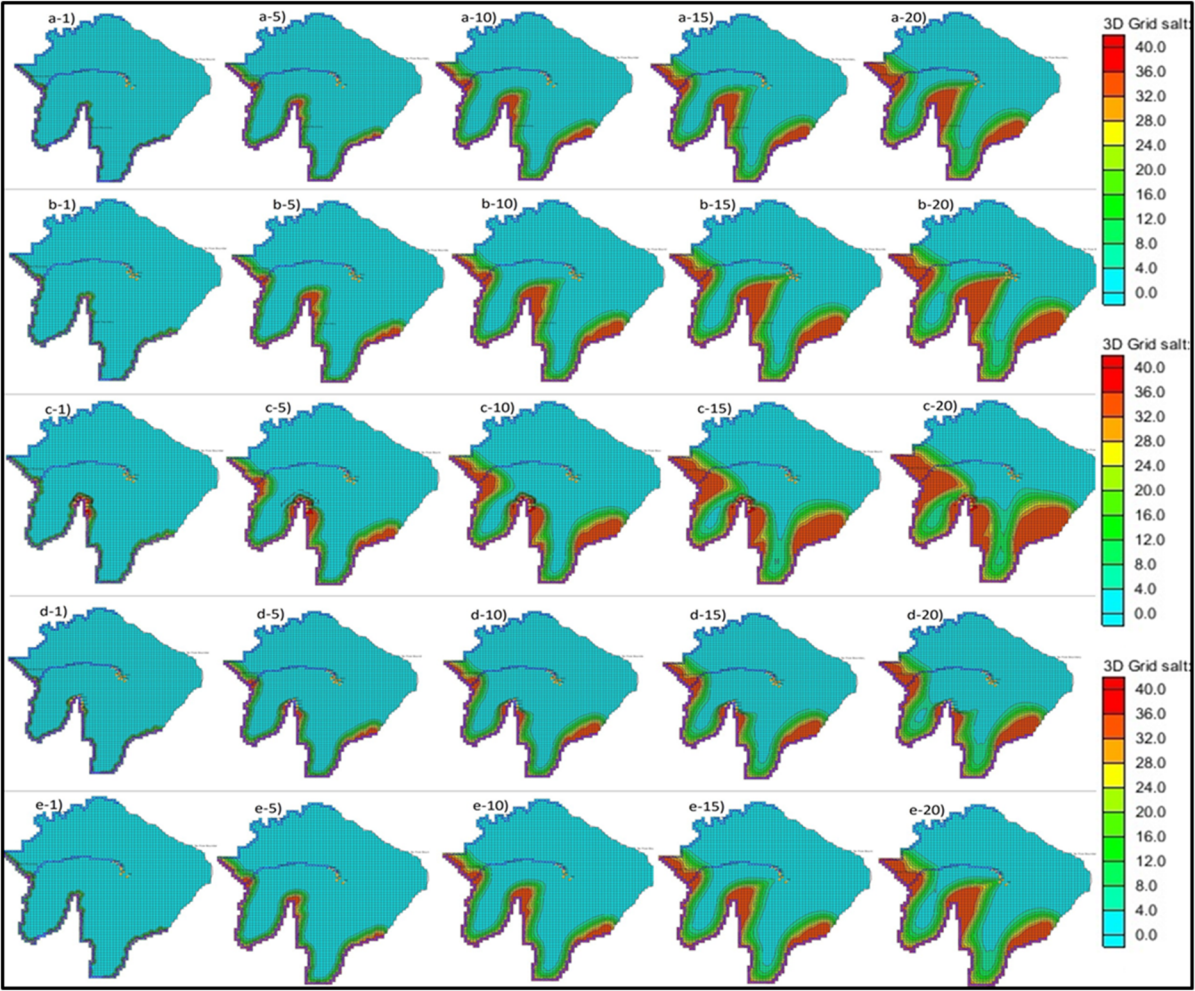
(a-1–a-20) shows the plan view of the simulated results of saltwater intrusion for the base case; (b-1–b-20) increasing the pumping and number of pumping wells (scenario 1); (c-1–c-20) addition of barrier wells (scenario 2); (d-1–d-20) addition of injection well plus barrier wells (scenario 3); (e-1–e-20) sea level rise (scenario 4). The numbers 1, 5, 10, 15, and 20 represent the time steps 1 year, 5 years, 10 years, 15 years, and 20 years, respectively

**Fig. 5:**
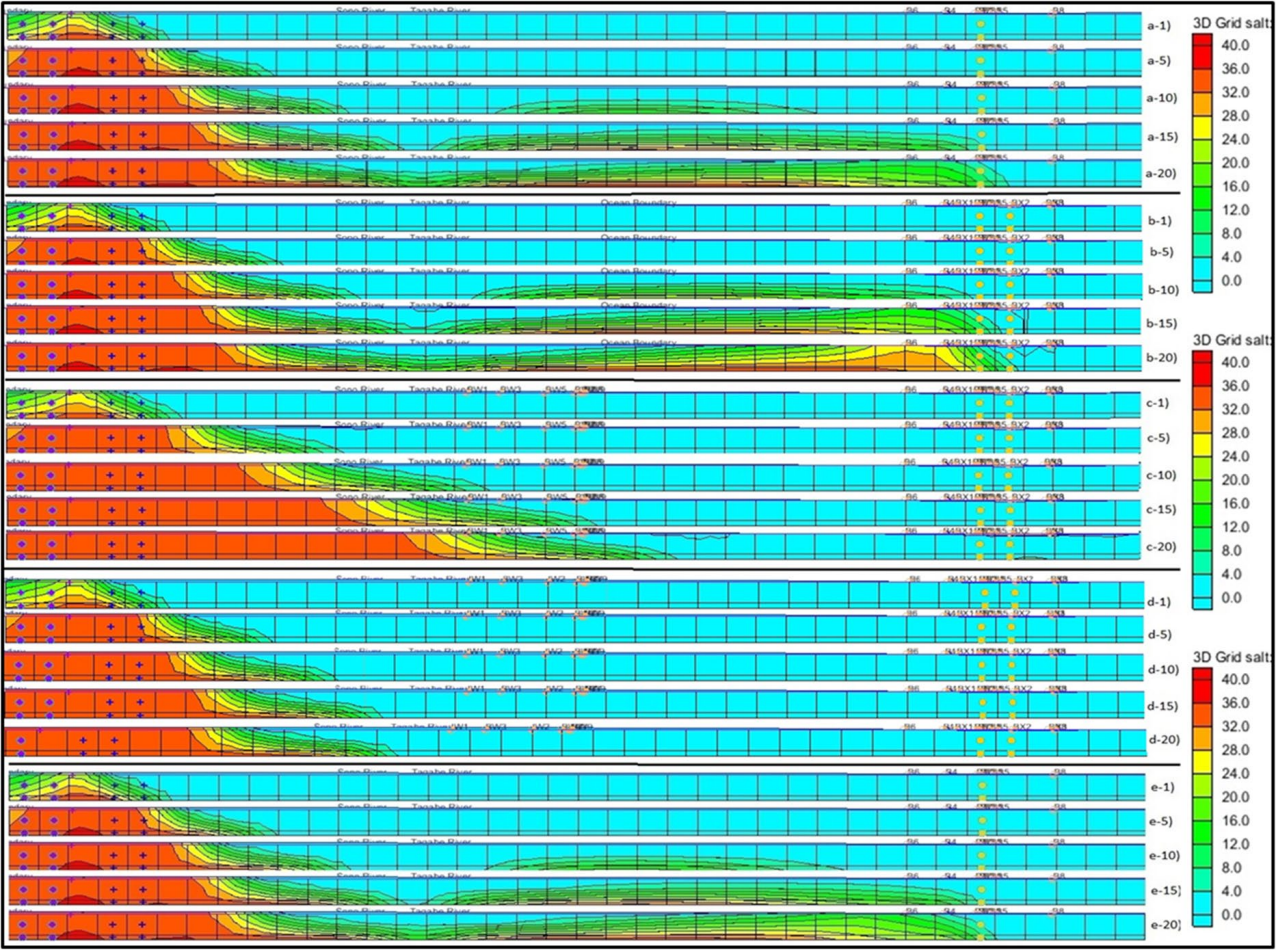
(a-1–a-20) shows the front view of the simulated results of saltwater intrusion for the base case; (b-1–b-20) increasing the pumping and number of pumping wells (scenario 1); (c-1–c-20) addition of barrier wells (scenario 2); (d-1–d-20) addition of injection well plus barrier wells (scenario 3); (e-1–e-20) sea level rise (scenario 4). The numbers 1, 5, 10, 15, and 20 represent the time steps 1 year, 5 years, 10 years, 15 years, and 20 years, respectively

Tagabe coastal aquifer has eight operational boreholes which supply the water to the capital city of Vanuatu. Based on the current field pumping data, MODFLOW interpolated the transient pumping data for a 20-year period which was used to predict the SWI in Tagabe coastal aquifer. The calibrated model was simulated for 20 years, and the SWI was recorded. The plan view of the model domain for the base case is shown in Fig. 4 (a-1–a-20). Five different time step results were recorded after the simulation. The front view of the base case simulation results was also recorded, as shown in Fig. 5 (a-1–a-20). The numbers 1-, 5-, 10-, 15-, and 20- indicate 1-year, 5-year, 10-year, 15-year, and 20-year time steps. The contours indicate the SWI into Tagabe coastal aquifers. Red indicates > 30 kg/m^3^ salt concentration, whereas light blue indicates ∼ 0 kg/m^3^ salt concentration.

### Scenario 1: increased population and pumping rates

**Fig. 6:**
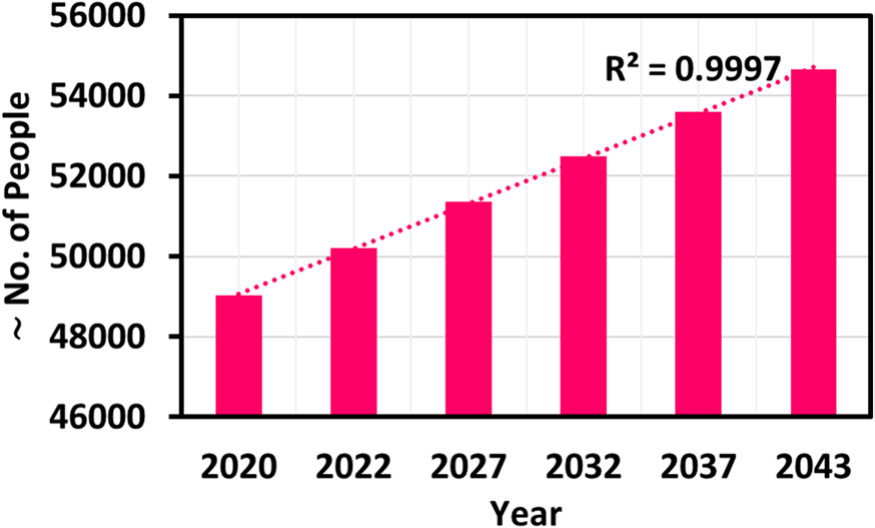
Projected population of Port Vila, Vanuatu

Population growth is inevitable in the future. A higher population will lead to higher water demand in all sectors. Since Tagabe coastal aquifer is the only water supplier in Port Vila, we must carefully consider the uncertainties while predicting the SWI rates. The projected population growth rates of Port Vila for 20 years are given in Fig. 6. The projections were made based on the live population forecasts given by the World Population Review (WPR) (WPR, 2023). As per WPR, the population of Vanuatu will grow by 2.39%, 2.31%, 2.2%, 2.11%, and 1.98% for the years 2022, 2027, 2032, 2037, and 2043, respectively. Based on these projections and using the actual population of Port Vila from City Population (CP) for the year 2020 (CP, 2023), we found that by 2043, the population of Port Vila will be approximately 54,668. The projected population till 2043 is an increase of ∼10% compared to 2020.

Moreover, we increased our transient pumping rates of all boreholes by 10% based on population growth. Also, we added three more boreholes to cater to the higher demand and marked them as BX1, BX2, and BX3, as shown in Fig. 2. The transient pumping rates similar to bores 7 and 8 were assigned to the three new boreholes. The changes made in the conceptual model were mapped to MODFLOW and MT3DMS then SEAWAT was initiated. The plan view and front view of the simulated SWIs of this scenario for 5 time steps are shown in Fig. 4 (b-1–b-20) and Fig. 5 (b-1–b-20), respectively. The results indicated that the extent of SWI has further contaminated the Tagabe freshwater aquifer compared with the base case. The contours in these figures also indicated that increasing the pumping rates and the number of boreholes further increases the SWI.

**Fig. 7:**
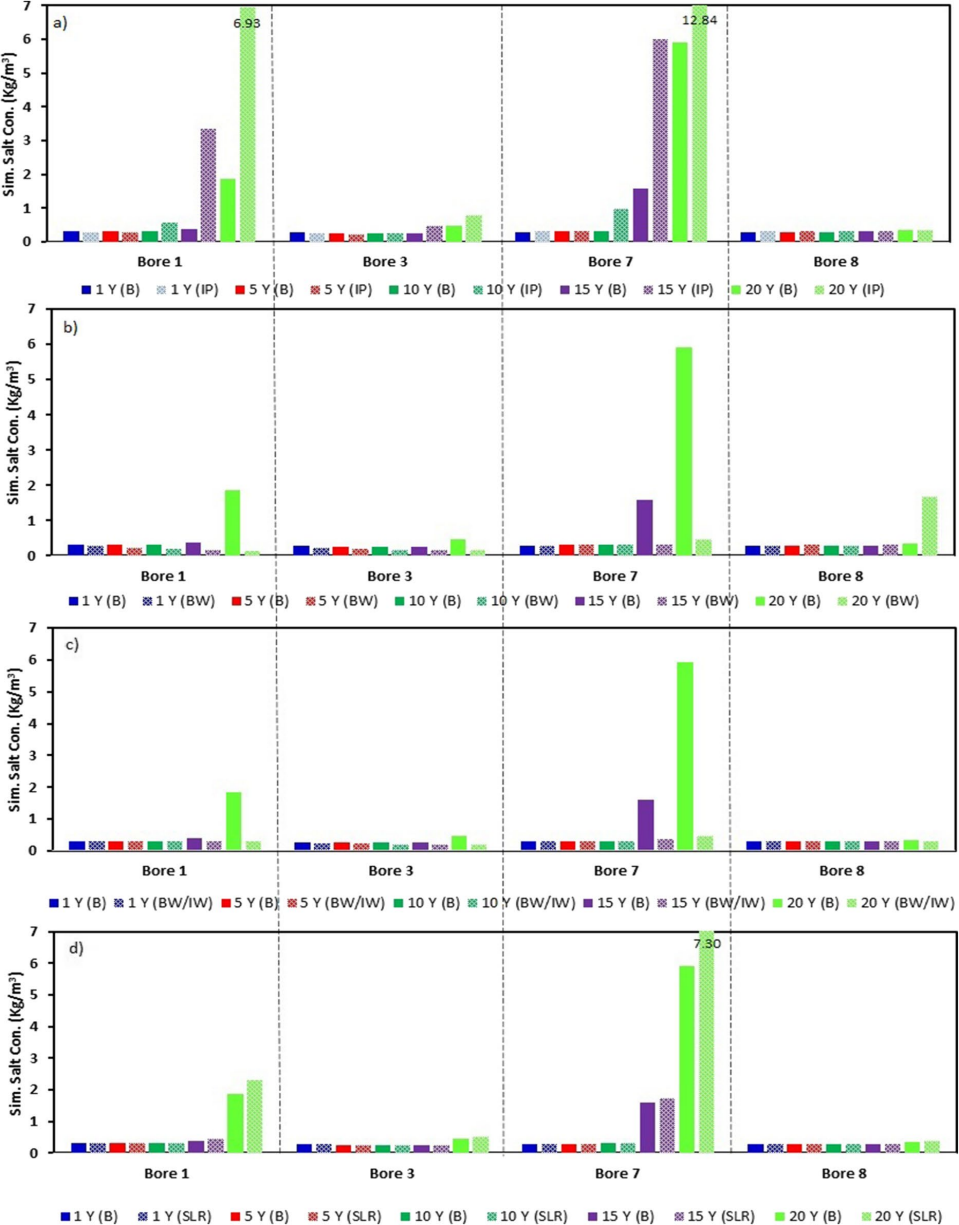
**a** shows the comparison of simulated salt concentrations for different boreholes for the base case and the increased pumping and the number of pumping wells (scenario 1); **b** shows the comparison of simulated salt concentrations for different boreholes for the base case the addition of barrier wells (scenario 2); **c** shows the comparison of simulated salt concentrations for different boreholes for the base case the addition of injection well plus barrier wells (scenario 3); **d** shows the comparison of simulated salt concentrations for different boreholes for the base case and the sea level rise (scenario 4)

Moreover, to further look at the exact amount of changes in the salt concentration, we recorded the salt concentrations for bores 1, 3, 7, and 8 for 1-year, 5-year, 10-year, 15-year, and 20-year time steps. Figure 7a compares the salt concentration of different time steps and bores for the base case and scenario 1. Not many changes were shown for the first two time steps. However, the last three time steps did show some significant changes. The percentage increase in salt concentration from base case to scenario 1 for bore 1 during the time steps of 10, 15, and 20 years are 86.34%, 759.32%, and 273.75%, respectively. Moreover, the percentage increase for bore 7 at 3 time steps as bore 1 was 209.79%, 277.78%, and 117.05%, respectively. Bores 3 and 8 did not show significant changes compared to the base case, which could have been the effect of transient and automatic pumping data interpolation in MODFLOW.

### Scenario 2: addition of barrier wells

Using the same parameters and boundary conditions from scenario 1, we added six barrier wells (BWs) to prevent the SWI from encroaching into the model domain. Based on the initial simulations, the BWs were systematically placed near the coast to prevent the upconing of the saline water. The BWs acted as a barrier in preventing the saltwater from moving towards the landward side and contaminating the freshwater, hence the name. The plan view of the simulated results for this scenario is shown in Fig. 4 (c-1–c-20). From the results, the extent of encroachment has significantly decreased when compared with the base case and scenario 1. However, the SWI from the southeast side of the model shows an increment for all 5 time steps. The increment is shown because the BWs were not added near the southeast coast. However, adding BWs has prevented the saline water from reaching the freshwater wells, as shown in the plan view.

The front view of the study area is also used to show our results for SWI better after adding BWs, shown in Fig. 5 (c-1–c-20). The front view shows that the SWI has significantly reduced after adding the BWs. However, the salt concentration near the coast has increased vastly due to excessive pumping from BWs. This increase is shown as the toe length of SWI has increased. The increase in the salt concentration did not affect the freshwater bore or wells in the Tagabe area. The front view also shows that the SWI contour’s length and strength have halved for 10-, 15-, and 20-year time steps compared to the base case scenario.

Moreover, the salt concentrations at bores 1, 3, 7, and 8 were recorded for the 5 time steps as scenario 1. Figure 7b compares the salt concentrations for the four bores after adding the BWs and the base case. The bars in the graph indicate that the salt has significantly decreased for bores 1, 3, and 7. However, not many changes were shown for bore 8. The salt concentration for bore 1 was reduced by 9.55%, 22.96%, 36.58%, 56.11%, and 92.29% for 1-, 5-, 10-, 15-, and 20-year periods, respectively. Similarly, the salt concentrations for bore 3 of the five time steps were also reduced by 16.47%, 28.86%, 32.57%, 34.92%, and 64. 19%, respectively. The salt concentration of bore 7 at the 20-year time step was 5.92 kg/m^3^ for the base case, whereas after adding BWs, the salt concentration was reduced to 0.46 kg/m^3^, a decrease of 92.19%. The salt concentrations at bore 8 for 1-, 5-, 10-, 15-, and 20-year time steps for this scenario were increased by 0.25%, 0.97%, 0.35%, 2.26%, and 391.65%, respectively.

### Scenario 3: addition of barrier wells and injection wells

The injection wells (IWs) and BWs were added to the model domain as an SWI management technique for the Tagabe coastal aquifer. In scenario 2, we added six BWs, whereas, in this scenario, we replaced three BWs with IWs. Therefore, for this scenario, we have three BWs and three IWs. Transient pumping rates, lower than BW pumping, were assigned to the IWs. The model simulation was initiated, and the plan view of the simulated results is given in Fig. 4 (d-1–d-20). The results show significant changes as the SWI is reduced further compared to the base case and scenarios 1 and 2. The length of encroachments from all sides of the model domain, from the coast to the pumping locations, was reduced significantly.

Furthermore, Fig. 5 (d-1–d-20) shows the front view of the simulated salt concentrations after adding BWs and IWs. The length of salt contours has significantly decreased compared to the base case and scenarios 1 and 2. The intensity of salt concentration at the coast is also reduced by injecting freshwater. The front view shows the encroachment length is half the scenario 2, whereas it is a quarter of the base case and scenario 1. Adding IWs has significantly reduced SWI in the Tagabe coastal aquifer.

The salt concentrations for bores 1, 3, 7, and 8 were significantly reduced when compared with the base case, as indicated by the bars of Fig. 7c. The percentage decrease in salt concentration for bore 1 during 1-, 5-, 10-, 15-, and 20-year time steps are 6.36%, 8.62%, 6.16%, 22.93%, and 83.73%, respectively. The percentage decrease for bore 3 for the same respective time steps as bore 1 is 10.93%, 14.67%, 17.33%, 18.01%, and 54.29%, respectively. Similar decreasing trends were also noted for bore 7. However, the highest decrease in salt concentration was noted at bore 7 for the 20-year time step, with a decrease of 92.37% compared with the base case. Moreover, bore 8 has shown slight increments of salt concentrations during 1-, 5-, 10-, and 15-year time steps. The percentage increments for these four time steps were 0.78%, 0.43%, 0.08%, and 0.11%, respectively. However, the salt concentration decreased during the 20-year time step by 9.63% when compared with the base case.

### Scenario 4: sea level rise

**Fig. 8:**
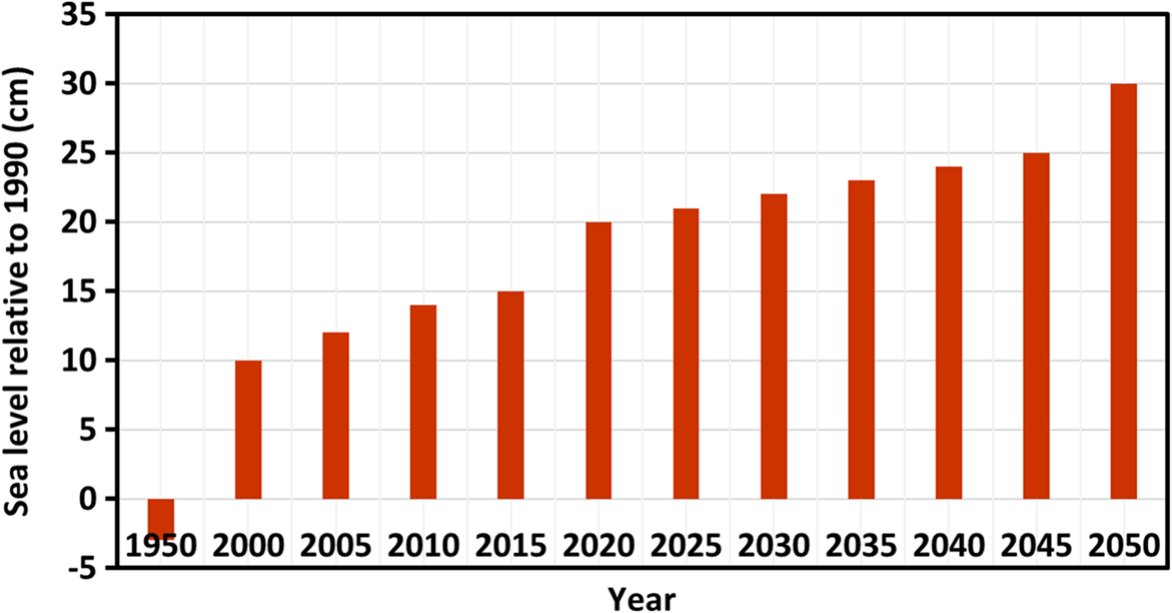
Projected sea level rise for Vanuatu, relative to 1990. Source: (CRCP, 2021)

The sea level rise (SLR) is one of the significant natural disasters in Vanuatu. The SLR could also be affecting the Tagabe freshwater aquifer. We modelled the SLR for Tagabe coastal aquifer to validate the previous statement, and the extent of SWI was noted. The projected SLR for Vanuatu, in general, is shown in Fig. 8. Based on the projections, the SLR for Vanuatu by 2043 will be approximately raised by 25 cm. Hence, we increased our coastal head boundaries gradually by 1.2 cm each year for 20 years until it reached 25 cm. Using this projected SLR, the model was simulated. The increase in head values for the coastal boundary indicated SLR. The plan view of the simulated results is shown in Fig. 4 (e-1–e-20). The boundary conditions and other initial conditions used for the simulation of SLR were used from the base case model design.

Moreover, to see the SLR effects further, we compiled the front view for 1-, 5-, 10-, 15-, and 20-year time steps as shown in Fig. 5 (e-1–e-20). The front-view results are also similar to the base case results. However, the contour and the salt concentrations for year 20 are higher, as indicated by colour changes. The more orange and yellow colours are noted in the contours indicating higher salt concentrations. From the front view the changes are less significant when compared with the base case. However, to see the effect of SLR on the intensity of SWI, we noted the salt concentration values, and the bar graph was plotted to compare the salt concentrations with the base case. The results are shown in Fig. 7d.

Using the bar graph, it was visible that SLR does have some impact on the water quality in Tagabe coastal aquifer. The percentage increase in salt concentration for bore 1 during 1-, 5-, 10-, 15-, and 20-year time steps is 0.07%, 0.19%, 0.36%, 14.99%, and 24.45%, respectively. Similarly, the percentage increase for the same time steps for bore 3 is 0.33%, 1.11%, 1.67%, 4.61%, and 16.07%, respectively. Moreover, the percentage increase in salt concentration for bore 7 is also noted for all time steps. The highest salinity increases for bore 7 were noted at a 20-year time step of 23.37% compared to the base case. The salt concentration changes for bore 8 were insignificant, except at the 20-year time step, which was around 13.28% compared to the base case scenario.

## Discussion

The SWI in Tagabe coastal aquifer was modelled using real field data. The simulation results indicated that SWI is currently affecting and will also affect freshwater resources in the future for Tagabe. Using the field data, the model was calibrated and validated. The calibrated model was used to test different scenarios that could help manage SWI in Tagabe coastal aquifer. The scenario-based sensitivity analysis for this research was the first for Vanuatu and any PICs. Based on the scenarios and sensitivity analysis, robust SWI management regulations can be drawn for Tagabe coastal aquifer.

Using hydraulic barriers like barrier wells (BWs) to abstract the saline water near the coast prevents the extent of SWI from contaminating the freshwater wells. Armanuos et al. (2020) effectively showed that using BWs near the saltwater side reduced the SWI rates or SWI length ratio. Abdoulhalik et al. (2017) also stated that using BWs reduced the SWI rates in coastal aquifers. Similarly, our results also indicated that using BWs near the coast positively affected the SWI in Tagabe coastal aquifer. The scenario that showed exciting results in minimising the extent of SWI in our model domain was using mixed BWs and IWs. Abd-Elaty et al. (2021) investigated the strategies for controlling the SWI due to sea level rise (SLR) and climate change for humid and hyper-arid regions. The authors reported that using recharge wells or IWs, brackish water abstraction, and desalination reduced the SWI rates. The authors suggested using mixed hydraulic barriers to manage SWI. Similar results were noted in our study.

Moreover, Yang et al. (2021) showed how to mitigate the SWI using pumping and injection strategies. The authors reported that IWs and PWs are more effective in managing SWI than using IWs or BWs alone. Our study also revealed that using the combination of hydraulic barriers like BWs and IWs helps to further reduce the extent of SWI in the Tagabe coastal aquifer. The hydraulic barriers implemented in this study showed that SWI could be managed. BWs and IWs alter the hydraulic gradient and prevent the upconing of the saline water. Werner et al. (2013) reported that the hydraulic barriers block the saline water encroachments by increasing the difference between the groundwater head and seawater head. Similarly, our results indicated that hydraulic barriers effectively block saline water from reaching freshwater wells.

However, physical barriers like subsurface dams and cutoff walls in controlling SWI are also widely used (e.g.Abdoulhalik et al., 2022; Kaleris & Ziogas, 2023; Luyun et al., 2011). These studies have investigated the effect of cutoff walls on SWI in coastal aquifers, and they reported that the use of cutoff walls is effective in managing SWI. However, this study is the first for Tagabe coastal aquifer, therefore only hydraulic barriers were used and modelled for the study area. The use of physical barriers in managing SWI for the Tagabe coastal aquifer will be added to our future scope of work. Moreover, cutoff walls physically prevent the SWI and it does not need any maintenance. However, the initial costs are high (Abdoulhalik et al., 2017). Cutoff walls could be used for Tagabe in the future.

Moreover, the use of BWs can also have some adverse effects on the management of SWI. Pool and Carrera (2010) studied the dynamics of negative hydraulic barriers to prevent SWI and reported that BWs sometimes abstract more freshwater, reducing the freshwater volume in aquifers. The disposal of abstracted saline water is an issue because it cannot be used anywhere unless its desalinated. Therefore, we proposed using the IWs along with BWs. The abstracted saline water can be desalinated and injected using IWs in Tagabe. If this process seems expensive and time-consuming, the abstracted saline water can be disposed of back into the ocean. Disposing saline water back into the ocean will not be costly because most of the population lives near the coast, and the freshwater wells are not far from the coast. Hence, a shorter length of pipes may be required to dispose of the saline water. If the abstracted saline water is disposed of in the ocean, the input for injection wells can be taken from freshwater sources like the Tagabe River or Sono River in Port Vila. Also, due to high rainfalls in Vanuatu, the rainwater could be harvested and injected backing to the aquifer to increase the aquifer volumes.

The hydraulic barriers proposed and modelled for Tagabe coastal aquifer would manage SWI and protect the freshwater wells from over-abstraction of groundwater. However, climate change and sea level rise (SLR) also affects the groundwater in Tagabe. Therefore, the SLR factor was considered and modelled for Tagabe coastal aquifer. The results indicated that the extent of SWI and the strength of salt concentrations in the freshwater aquifer have increased. Similar results were reported by Casillas-Trasvina et al. (2019) and Romanazzi et al. (2015) for a different study area. Moreover, the SLR by 25 cm was less significant, and fewer variations were noted in our study. The SLR and SWI simulation results were less significant because raising the hydraulic head by 25 cm has significantly less impact on the SWI rates. Loaiciga et al. (2011) and Sharan et al., (2023a, b) also reported that SLR by < 1 m has less effect on the SWI rates in coastal aquifers. However, the length and strength of SWI would be extensive if SLRs by > 1 m in the future. The SLR by > 1 m would be considered for the future scope of work for Tagabe coastal aquifer.

Based on the scenario testing and sensitivity analysis, the SWI management strategies for Tagabe coastal aquifer were proposed, as discussed above. Using the results from numerical modelling, the water resource managers, site owners, or governing bodies can draw up policies and regulations for using Tagabe groundwater. However, due to uncertainties and the variability of climate conditions in Vanuatu, we suggest that the policies and regulations be revised every year. Factoring the different boundary conditions, pumping patterns, and uncertainties, hydrogeologists can remodel and simulate new sets of SWI management strategies. However, this can be challenging and computationally extensive because numerical modelling is a complex and time-consuming exercise. Hence, using AI-based surrogate and ensemble models would be much easier, more efficient, and less computationally extensive to simulate SWI management strategies for coastal aquifers in Vanuatu and in PICs.

## Conclusions

The Pacific Island countries (PICs) groundwater resources are threatened by anthropogenic and natural factors. A small PIC, Vanuatu’s groundwater system, was studied using real field data from Tagabe coastal aquifer, which supplies freshwater in Port Vila. However, Tagabe is experiencing some salinity concentrations in the freshwater wells. Therefore, the primary aim of this study was to provide saltwater intrusion (SWI) management strategies for Tagabe coastal aquifer.

The SWI management strategies were developed based on numerical modelling results. This is the first study in Vanuatu whereby a numerical model was developed using the SEAWAT code. The developed model was calibrated and validated using the salinity data. The calibrated model was then used to test different scenarios that could effectively manage SWI. The sensitivity analysis revealed that using hydraulic barriers like barrier wells (BWs) and injection wells (IWs) significantly reduced the extent of SWI in Tagabe coastal aquifers. We found that using BWs and IWs in combination was the best technique for managing SWI.

Moreover, the sea level rise (SLR) was modelled to see how it affects the SWI in Tagabe coastal aquifer. It was found that SLR had some effects on SWI rates. The numerical simulation period for the study area was till 2043. Hence, the sea level was only raised by the projected level of 2043, which was < 30 cm. Therefore, the effect of SLR was not large. However, simulating the model with higher sea face boundary conditions and for larger stress periods would have a significant effect due to SLR. Moreover, uncertainties need to be catered for while forecasting future scenarios.

The management strategies proposed in this study can be used by the water managers, site owners, and governing bodies to draw up policies and regulations to manage the SWI rates in Tagabe coastal aquifer. These strategies will protect the groundwater resources from getting contaminated by SWI in the future. Moreover, this study can also benefit some stakeholders or bore drilling companies who want to harvest groundwater from the Tagabe coastal aquifer, as it will give them the benchmark of daily or yearly freshwater production rates without SWI.

The limitation of this study was the availability of quality groundwater data. Also, the hydraulic head values were not available that could have been used to calibrate the flow model. We had very limited pumping data and salinity data. Therefore, we were only able to calibrate the transport model. Furthermore, only one borelog was available, which we used to gather hydrogeological parameters and other ground stratigraphy.

## Recommendation and future scope of work

The recommendations and future work that we are proposing to carry out for Tagabe coastal aquifer are (1) setting up proper groundwater heads and water quality data monitoring sites in collaboration with the Government of Vanuatu, (2) developing AI-based modelling techniques, (3) using physical barriers in model to simulate SWI, and (4) rising the boundary conditions to factor higher (heads > 1 m) SLR.

## Data Availability

The datasets used and/or analysed during the current study are available from the corresponding author on reasonable request.
